# Injectable Self-Healing Adhesive pH-Responsive Hydrogels Accelerate Gastric Hemostasis and Wound Healing

**DOI:** 10.1007/s40820-020-00585-0

**Published:** 2021-02-27

**Authors:** Jiahui He, Zixi Zhang, Yutong Yang, Fenggang Ren, Jipeng Li, Shaojun Zhu, Feng Ma, Rongqian Wu, Yi Lv, Gang He, Baolin Guo, Dake Chu

**Affiliations:** 1 Department of Gastroenterology, The First Affiliated Hospital of Xi’an Jiaotong University, Xi’an, People’s Republic of China; 2 Frontier Institute of Science and Technology, and State Key Laboratory for Mechanical Behavior of Materials, Xi’an Jiaotong University, Xi’an, 710049 China; 3 Key Laboratory of Shaanxi Province for Craniofacial Precision Medicine Research, College of Stomatology, Xi’an Jiaotong University, Xi’an, 710049 People’s Republic of China; 4 Department of Dermatology, The First Affiliated Hospital of Xi’an Jiaotong University, Xi’an, 710049 People’s Republic of China; 5 National Local Joint Engineering Research Center for Precision Surgery and Regenerative Medicine and Surgical Engineering Research Center of Shaanxi Province, First Affiliated Hospital of Xi’an Jiaotong University, Xi’an, People’s Republic of China; 6 Department of Experimental Surgery, Xijing Hospital, Fourth Military Medical University, Xi’an, People’s Republic of China; 7 Department of Pathology, Tangdu Hospital, Fourth Military Medical University, Xi’an, People’s Republic of China

**Keywords:** Injectable self-healing hydrogel, Adhesive hydrogel, Gastric hemostasis, Gastric wound healing, Endoscopic treatment

## Abstract

Endoscopic mucosal resection (EMR) and endoscopic submucosal dissection (ESD) are well-established therapeutics for gastrointestinal neoplasias, but complications after EMR/ESD, including bleeding and perforation, result in additional treatment morbidity and even threaten the lives of patients. Thus, designing biomaterials to treat gastric bleeding and wound healing after endoscopic treatment is highly desired and remains a challenge. Herein, a series of injectable pH-responsive self-healing adhesive hydrogels based on acryloyl-6-aminocaproic acid (AA) and AA-g-N-hydroxysuccinimide (AA-NHS) were developed, and their great potential as endoscopic sprayable bioadhesive materials to efficiently stop hemorrhage and promote the wound healing process was further demonstrated in a swine gastric hemorrhage/wound model. The hydrogels showed a suitable gelation time, an autonomous and efficient self-healing capacity, hemostatic properties, and good biocompatibility. With the introduction of AA-NHS as a micro-cross-linker, the hydrogels exhibited enhanced adhesive strength. A swine gastric hemorrhage in vivo model demonstrated that the hydrogels showed good hemostatic performance by stopping acute arterial bleeding and preventing delayed bleeding. A gastric wound model indicated that the hydrogels showed excellent treatment effects with significantly enhanced wound healing with type I collagen deposition, α-SMA expression, and blood vessel formation. These injectable self-healing adhesive hydrogels exhibited great potential to treat gastric wounds after endoscopic treatment.

**Supplementary Information:**

The online version of this article (10.1007/s40820-020-00585-0) contains supplementary material, which is available to authorized users.

## Highlights

- A series of novel injectable pH-responsive self-healing hydrogels with enhanced adhesive strength were prepared.
- The hydrogels showed good gastric hemostasis property in a swine gastric hemorrhage model.
- The hydrogels greatly enhanced gastric wound healing in a swine gastric wound model.

**Supplementary Information:**

The online version of this article (10.1007/s40820-020-00585-0) contains supplementary material, which is available to authorized users.

## Graphical abstract

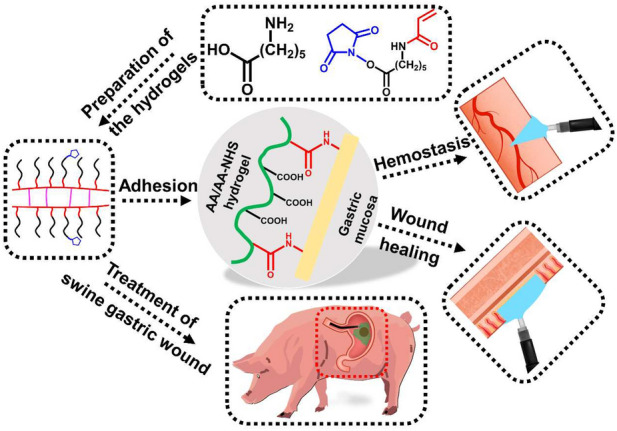

## Introduction

Gastrointestinal cancer is one of the most common malignancies worldwide [1]. Although surgical treatment, chemotherapy, and immunotherapy for gastrointestinal cancer have advanced in recent decades, due to its invasive and metastatic nature, gastrointestinal cancer remains a leading cause of cancer-related death [2, 3]. With the progression of endoscopic techniques, the screening for and early diagnosis of gastrointestinal neoplasias, including polyps and precancerous lesions, have shed light on early treatment [4]. Endoscopic mucosal resection (EMR) and endoscopic submucosal dissection (ESD) are well-established therapeutics that are widely used methods for gastrointestinal neoplasias [5]. These endoscopic techniques possess several advantages, including being less invasive than surgery, having a curative resection effect and retaining the physiological gastrointestinal structure. Although endoscopic treatment techniques and instruments have been improving, EMR and ESD of the mucosal layer are associated with intra- and postprocedural complications such as bleeding and perforation [6, 7]. Additionally, postprocedure bleeding can develop as late as 2 weeks after treatment [8]. These complications can result in extended hospitalization, additional treatment, morbidity and potentially life-threatening situations. Moreover, infection and physicochemical stimulation aggravate chronic inflammation after endoscopic treatment and delay extracellular matrix (ECM) degradation and the wound healing process. Faced with the great challenge of rapid hemorrhage after endoscopic treatment, a variety of commercial hemostatic spray powders that have the ability to spray toward the lesion through a catheter have been developed in recent years and have shown a certain therapeutic potential to promote gastric wound healing and hemostasis [9–11]. However, the hemostatic effects of these powders usually last for only a short time (up to one day) because they have low adhesive strength to the wound and dissolve quickly. Furthermore, the powder is often delivered to the lesion by a catheter, which leads to indiscriminate dispersion of the powder from the tip of the catheter that obscures the field of vision, which places an additional burden on the operative process [12]. Despite numerous studies focusing on ideal wound dressing materials that could accelerate coagulation, tissue formation, angiogenesis, and re-epithelialization, there are currently no available endoscopic sprayable materials that efficiently promote gastric wound healing after endoscopic treatment.

Fortunately, in situ-formed injectable hydrogels (ISFIHs) can be injected into wounds without gel fragmentation and then integrated as bulk gels to heal the wound [13, 14]. Specifically, the ISFIH is initially a liquid at room temperature and has a pre‐gelling fluidity that can be applied to any defect or cavity with minimal invasiveness [15–17]. After undergoing transitions within a short time or in response to pH/temperature changes, the hydrogel will then be formed in situ and can rapidly cross-link with the tissues around the wound site to act as a bioadhesive material to bind tissues together, sealing leaks and stopping unwanted bleeding [18–25]. In addition, ISFIHs can be tailored to mimic the physicochemical properties of human tissues and promote the wound healing process [26, 27]. Given these unique characteristics, ISFIHs have attracted increasing attention in recent years and are widely used in various biomedical applications [14, 28, 29]. However, until now, there have been no reports on ISFIHs for treating gastric bleeding and wound healing after endoscopic treatment.

Long-term applications of ISFIHs in gastric wound healing are necessary, as bleeding after EMR/ESD procedures can even develop as late as 2 weeks after surgery [8]. Therefore, the basic demands of the sprayed hydrogels around the wound site are that they need to be able to withstand long-term mechanical forces from the stomach (approximately 1000 times per day, 5–10 kPa each time) and self-heal after the external mechanical force [30] because tension caused by gastric peristalsis, dilatation, and contraction would damage the hydrogels. Thus, hydrogels with the ability to rapidly self-heal and automatically repair themselves after damage could extend their service life after application to wound sites [31]. In addition, the incidence of bleeding and perforation after surgery is high in the stomach due to the gastric acid environment and loss of the mucus-bicarbonate and mucosal barriers [32]. Thus, an ideal wound dressing should possess efficient and stable adhesive behavior to strongly adhere to the wound site, act as a barrier to protect the wound from the external environment and provide a suitable microenvironment to accelerate the healing process of the wound after endoscopic treatment [33, 34]. Additionally, the stable and long-term adhesive properties of hydrogels can prevent their detachment from the wound site and lead to improved and longer-lasting wound protection from gastric acid [35, 36]. However, until now, there have been no reports on the design of hydrogels with multifunctional properties that integrate injectability, an autonomous self-healing capacity, and efficient and stable adhesive behavior in acidic conditions to treat gastric bleeding and wound healing after endoscopic treatment.

In this study, we developed a series of multifunctional hydrogels by the free-radical polymerization of the monomers 6-aminocaproic acid (AA) and AA-g-N-hydroxysuccinimide (AA-NHS), and this series of hydrogels with injectable self-healing and adhesive properties can be sprayed through a gastroscope to act as a sealant/adhesive/hemostat to rapidly stop bleeding and accelerate gastric wound healing. The in vivo hemostasis performance and wound healing behavior of the AA/AA-NHS hydrogels were investigated with a swine gastric hemorrhage/wound model. The hemostatic effects, wound healing results, and histomorphological evaluations suggested that the therapeutic efficacy of the AA/AA-NHS10 hydrogel-treated group was more effective than that of the proton pump inhibitor (PPI)-treated and control groups. AA/AA-NHS hydrogels were prepared by mixing the precursor solution of AA and AA-NHS with N,N′-methylenebisacrylamide (BIS) as a cross-linker to form the hydrogel network. Hydrogels containing AA-NHS showed remarkable adhesive strength on the substrates of the porcine stomach because NHS-activated AA could act as a novel micro-cross-linker to covalently cross-link with the –NH_2_ groups of cell membranes. The chemical structure, injectability, rheology, self-healing behavior, morphology, swelling behavior, adhesiveness, in vitro/in vivo biocompatibility, and hemostasis and wound healing therapeutic efficacy of the AA/AA-NHS hydrogels were systematically characterized. All of the results suggested that these injectable self-healing adhesive hydrogel wound dressings exhibit great clinical potential for rapid hemostasis and the promotion of gastric wound healing after endoscopic treatment.

## Experimental Section

### Preparation of the AA/AA-NHS Hydrogels

AA and AA-NHS were synthesized according to references [37, 38], the detailed procedures are shown in the Supporting Information (SI). AA/AA-NHS hydrogels were prepared based on the free-radical polymerization of the AA monomer and AA-NHS at room temperature. APS (50 mg mL^−1^) was used as the initiator and tetramethylethylenediamine (TEMED, 10 µL mL^−1^) was used as an accelerator for gelation. The hydrogels with AA-NHS contents of 0, 5, 10, 15, and 20 mg mL^−1^ were named AA/AA-NHS0, AA/AA-NHS5, AA/AA-NHS10, AA/AA-NHS15, and AA/AA-NHS20, respectively. Table S1 shows the parameters and gelation times of the hydrogels.

### Characterization

Fourier transform infrared (FT‐IR) spectroscopy, nuclear magnetic resonance (NMR) spectroscopy, field emission scanning electron microscopy (SEM), gelation time tests, swelling ratios, and degradation tests were conducted to investigate the physical and chemical characteristics of the AA monomer, AA-NHS, and the AA/AA-NHS hydrogels. The SI shows the detailed procedures of these tests.

### Mechanical Behavior of AA/AA-NHS Hydrogels

The detailed procedures for evaluating the mechanical behavior of AA/AA-NHS hydrogels which include rheological behavior, self-healing properties [14, 39], and adhesive strength [27] are available in SI.

### Biocompatibility Assessments and Hemostatic Performance of the AA/AA-NHS Hydrogels

The biocompatibility assessments by employing a hemolysis activity assay [40], cytocompatibility assessment [41], and the host inflammatory response tests. The hemostatic performance of AA/AA-NHS hydrogels was evaluated by employing a mouse liver trauma model, a mouse liver incision model, and a mouse tail amputation model (Kunming mice, 30–35 g, female). The detailed procedures for the biocompatibility and hemostatic test are shown in SI.

### Hemostatic Performance and Wound Healing Behavior of the AA/AA-NHS Hydrogels in a Swine Gastric Wound Model

The AA/AA-NHS hydrogel gastric hemorrhage model experiment was performed at the animal experiment center of Xi’an Jiaotong University. To investigate the hemostatic effects and healing acceleration effects of the AA/AA-NHS hydrogels, two experiments were conducted separately. Twelve swine with body weights of 35 ± 2.1 kg (32.9–37.0 kg) were utilized in each experiment. The SI shows the detailed procedures for the hemostatic performance and wound healing behavior tests. All experimental protocols were approved by the ethics committee of Xi’an Jiaotong University.

### Statistical Analysis

All results were analyzed statistically and expressed as the mean ± standard deviation (SD). One-way ANOVA was used to determine significant differences, followed by Bonferroni’s post hoc test for multiple comparisons with SPSS, version 24 (IBM). Differences were considered significant if *P* < 0.05.

## Results and Discussion

### Synthesis and Characterization of the Injectable Self-healing Adhesive Hydrogels

In this study, a series of injectable self-healing adhesive hydrogels were prepared by the free-radical polymerization of AA precursors and AA-NHS, and these hydrogels showed stable mechanical properties, good biocompatibility, and hemostatic ability. Importantly, it is worth mentioning that these hydrogels possessed good adhesiveness to the porcine stomach and had great potential for treatment in gastric bleeding models.

**Fig. 1:**
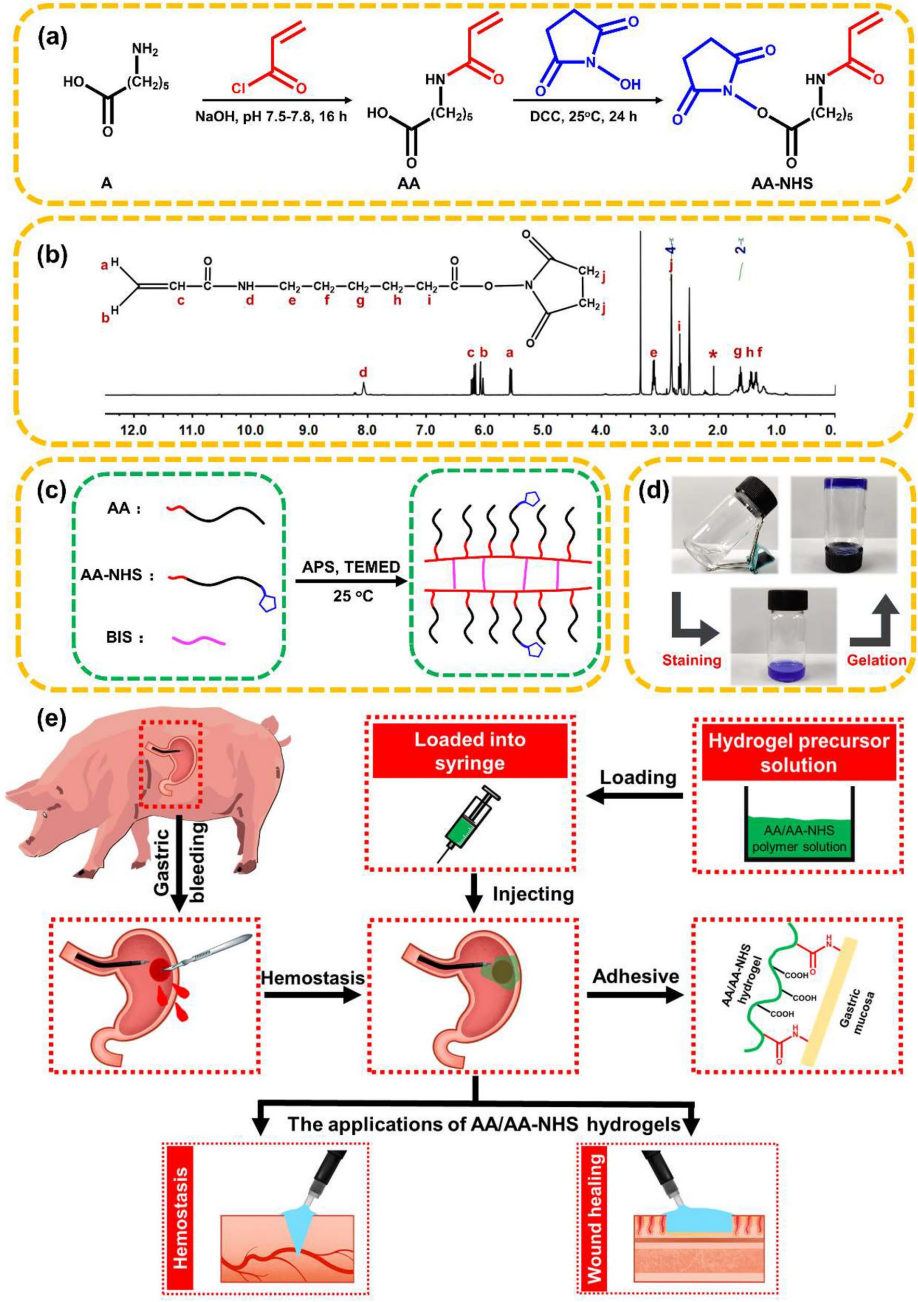
Schematic representation of AA/AA-NHS hydrogel synthesis. **a** Synthesis of AA and AA-NHS ester copolymers. **b** ^1^H NMR spectrum of AA-NHS. **c** Preparation and network of the AA/AA-NHS hydrogel. **d** Photographs of the AA/AA-NHS hydrogel polymer solution before and after crosslinking. The solution was stained with crystal violet. **e** The applications of AA/AA-NHS hydrogels for hemostasis and wound healing

Figure 1 shows a schematic representation of the injectable adhesive hydrogels. AA was first synthesized by grafting acryloyl chloride onto the backbone of A, and then AA-NHS was produced by esterification of the –COOH group of AA and the –OH group of NHS (Fig. 1a). The chemical structures of AA-NHS and AA were confirmed by ^1^H NMR and FT-IR (Figs. 1b and S1, S2). In the spectrum of AA (Fig. S1), the characteristic peak of the proton at 12.00 ppm was assigned to the carboxyl group and the integral intensity ratio of 1:2, which corresponds to the number of protons in acryloyl chloride (a-proton) and A (g-proton), suggesting that each A monomer chain was grafted with acryloyl chloride. In the spectrum of AA-NHS (Fig. 1b), the carboxyl group proton peak disappeared, while a newly formed peak that appeared at 2.81 ppm corresponded to the protons of NHS. Additionally, the signal from the j-proton of AA-NHS showed an integral intensity ratio of 4:2 compared to that of the g-proton, which illustrated that the carboxyl groups of AA were completely esterified with the hydroxyl groups of NHS and that the NHS-activated AA ester (AA-NHS) was successfully synthesized. AA/AA-NHS hydrogels were then synthesized by mixing the AA and AA-NHS solutions together at room temperature with BIS as a cross-linker to form a network of hydrogels via free-radical polymerization (Fig. 1c, d). A previous study showed that AA-based adhesive hydrogels exhibited good adhesion to the gastric mucosa and that the adhesion was strong enough to support the weight of the hydrogel [38]. Moreover, the stomach is an acidic environment, which is beneficial for the protonation of most of the terminal carboxyl groups of AA in the hydrogels and allows these groups to form hydrogen bonds with other terminal carboxyl or amide groups within the hydrogel for rapid self-healing. NHS-activated AA can be used to efficiently covalently cross-link with the –NH_2_ groups of the cell membranes in the stomach, and then the adhesion of the hydrogels to the gastric mucosa can be significantly improved through the newly formed –CO–NH– bond (Fig. 1e). Taken together, the efficient and stable adhesion properties of the hydrogel and the rapid and robust self-healing behavior in an acidic environment broaden the applications of these hydrogels to be used as injectable self-healing sealants to seal deep and noncompressible wound bleeding (Fig. 1e). It is worth mentioning that these hydrogels show suitable gelation time (Table S1) and can be administered in a more convenient and simplified condition that is suitable for the first aid use of severe gastric hemorrhage in emergency situations. Specifically, the AA/AA-NHS hydrogel precursor solution exhibits a suitable gelation time (< 9 min), indicating that the hydrogel polymer solution can pre-polymerize for 3 min and then remain injectable after transfer to a 23-gauge endoscopic injection needle that is widely used in clinical application [42, 43]. Movie S1 shows that the hydrogel can be extruded through a 23-gauge needle without clogging after polymerization for approximately 3 min, and then the polymer solution will immediately gel after contacting the acidic solution. Thus, the prepolymerized hydrogel precursor solution can be injected into the submucosa of the posterior wall of the gastric body through the endoscopic injection needle to stop hemorrhage and promote the healing process in a gastric wound.

### Rheological Properties and Self-healing Behavior of the Hydrogels

**Fig. 2:**
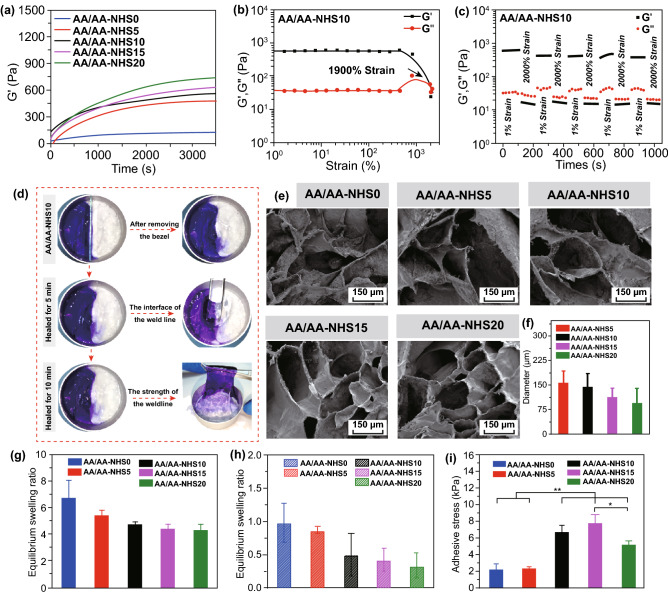
**a** Rheological behavior of AA/AA-NHS hydrogels. **b** *Gʹ* and *G*ʺ of the AA/AA-NHS10 hydrogel in the strain sweep test. **c** The rheological properties of the AA/AA-NHS10 hydrogels when the alternate step strain was switched from 1 to 2000%. **d** The macroscopic self-healing test of the AA/AA-NHS10 hydrogel. The hydrogel on the left was stained with crystal violet. **e** SEM images and **f** the pore size of the AA/AA-NHS hydrogels. Swelling behavior of AA/AA-NHS hydrogels at pH values of **g** 7.4 and **h** 2.0. **i** Adhesive strength of the AA/AA-NHS hydrogels on the substrates of the porcine stomach. *P < 0.05, **P < 0.01

To describe the effect of the AA-NHS content on the mechanical properties of these series of AA/AA-NHS hydrogels, the storage modulus (*G*ʹ) and loss modulus (*G*ʺ) of AA-NHS-containing hydrogels at a fixed strain of 1% were detected with a rheometer, and the temperature was set to 37 °C over the whole test to simulate body temperature. As shown in Fig. 2a, the AA-NHS-containing hydrogel groups exhibited relatively stable mechanical properties, and the *G*ʹ increased from 490 (AA/AA-NHS5) to 752 Pa (AA/AA-NHS20) as the AA-NHS content increased. In addition, the AA/AA-NHS0 hydrogel showed the lowest *G*ʹ (130 Pa) among all this series of AA-NHS-containing injectable hydrogels.

Considering the applications of these hydrogels in gastric bleeding and the possible damage of the hydrogels caused by gastric motility, the self-healing behavior of these hydrogels was further detected to evaluate whether the hydrogels can maintain their integrity after application of an external mechanical force. The rheological recovery test was employed to evaluate the self-healing capacity of the hydrogel, and the AA/AA-NHS10 hydrogel was chosen as an example. The result of the strain amplitude sweep in Fig. 2b indicates that the intersection point between *G*ʹ and *G*ʺ was 1900%, which means that AA/AA-NHS10 can withstand a large external mechanical while maintaining their integrity, and that the hydrogel network collapsed above this critical point (1900%). After this point, with an increase in the amplitude strain, the collapse of the AA/AA-NHS10 hydrogel network was further revealed (*G*ʹ < *G*ʺ). Next, the breakup and healing ability of the AA/AA-NHS10 hydrogel were investigated by conducting a strain amplitude sweep test at the predetermined time of 100 s (Fig. 2c). The value of *G*ʹ was significantly higher than that of *G*ʺ at the beginning, suggesting that the hydrogel network was stable. When the dynamic strain returned to 2000%, the value of *G*ʹ decreased from 600 to 16 Pa and was lower than the value of *G*ʺ, indicating that the hydrogel network collapsed. When the AA/AA-NHS10 hydrogel was subjected to a strain of 1%, *G*ʹ and *G*ʺ instantaneously almost returned to their initial values. Furthermore, the strain amplitude sweep test showed that the healing ability of the hydrogel was totally reversible and reproducible during the cyclic tests (*n* = 5).

A macroscopic self-healing test was further performed to demonstrate the healing behavior of the hydrogels (Fig. 2d). Four milliliters of crystal violet-stained AA/AA-NHS10 hydrogels was injected to the left side of a circular mold. Healing occurred instantly after the bezel was removed (no obvious boundary was observed). After 5 min of healing, the resultant hydrogels maintained their integrity, and the interface of the weld line did not show any tears, even after external force with forceps. Thus, the resulting healed hydrogels can sustain large deformations after 10 min of healing, which confirmed the excellent self-healing efficiency of the AA/AA-NHS10 hydrogels.

The efficient and repeatable self-healing properties of the hydrogel were attributed to the multiple interactions within the hydrogel matrix. The terminal carboxyl groups in AA were mostly protonated allowing hydrogen bonds to form with other terminal carboxyl groups across the interface, which is conducive for hydrogel self-healing. In addition, the side chains in the AA monomer are sufficiently flexible and long, which facilitates the –COOH functional groups on the interface to contact each other and mediate the synergistic effect of hydrogen bonds [38]. Hydrophobic interactions of the AA-NHS esters in aqueous media also facilitate the self-healing of AA/AA-NHS hydrogels because AA-NHS is a hydrophobic monomer, and hydrophobic interactions (a reversible noncovalent interaction) exist among these groups, which are conducive for reforming of the hydrogel network [15, 44–46]. All of these factors lead to the hydrogels possessing good self-healing behavior and high self-healing efficiency.

### Morphology, Swelling Ratio, and Adhesive Properties of the Hydrogels

The pH of the gastrointestinal tract varies significantly, from highly acidic (pH 1–3) in the stomach to neutral or weakly alkaline in the duodenum, jejunum, and ileum (pH 6–7.5) [47, 48]. Considering that the application of the hydrogels is for gastric wound bleeding and accelerated wound healing after endoscopic treatment, the morphology of AA/AA-NHS hydrogels was detected by immersing the bulk hydrogels in artificial gastric juice (pH 2.0) in sealed vials. The images in Fig. 2e show interconnected and uniform AA/AA-NHS hydrogel microstructures. The pore diameter of the hydrogel decreased from 156.7 to 145.1, 113.6, and 95.4 μm for AA/AA-NHS5, AA/AA-NHS10, AA/AA-NHS15, and AA/AA-NHS20, respectively (Fig. 2f). The equilibrated swelling ratios (ESRs) of the hydrogels were evaluated by immersing the hydrogels in PBS (pH 7.4) and in solution at pH 2.0 at 37 °C. All the hydrogels swelled sharply, with ESRs from 4.3 to 6.7 at pH 7.4 and ESRs from 0.3 to 0.9 at pH 2.0 (Fig. 2g, h). This is because the carboxyl groups in a low pH solution will form intramolecular hydrogen bonds, which will decrease the swelling behavior of the hydrogel [38]. The hydrogels exhibited a low swelling ratio in the acidic environment, which is beneficial for maintaining a stable network when used for gastric wounds. Besides, in vitro degradation tests suggested that AA/AA-NHS hydrogels will not be significantly degraded during the service period (Fig. S3), which can provide long-lasting and effective protection for the gastric bleeding site that can reduce the delayed bleeding caused by acid-corrosive injury and provide a suitable microenvironment to accelerate wound healing [8, 49].

Compared with the traditional hemostatic method, the hemostatic mechanism of hydrogels mainly relies on the good adhesiveness that can adhere to wound sites with a short gelation time. Afterward, the adhesion layer formed on the wound site can act as a physical barrier to prevent blood outflow [50]. Thus, the adhesive strength of AA/AA-NHS hydrogels was investigated by performing lap shear strength experiments. Considering that the application of these hydrogels is for acute gastric mucosal hemorrhage, the adhesive strength of the AA/AA-NHS hydrogels was detected by applying the hydrogels to the substrates of the porcine stomach. The lowest adhesive strength was observed in the AA/AA-NHS0 (2.19 kPa) and AA/AA-NHS5 (2.32 kPa) groups. As the concentration of AA-NHS increased, the adhesive strength of the hydrogel showed an increasing tendency, and the greatest adhesive strength was observed for the AA/AA-NHS10 (6.63 kPa) and AA/AA-NHS15 (7.96 kPa) hydrogels. This result was due to the presence of the NHS-activated AA, which could act as a novel micro-cross-linker to covalently cross-link with the –NH_2_ groups of the cell membranes covering the wound site, significantly improving the adhesive strength by the newly formed amide bond (–CO–NH–) [51]. However, the adhesive strength decreased to 5.16 kPa once the content of AA-NHS reached 20 mg mL^−1^ (Fig. 2i). This may be a result of the adhesion performance of the AA/AA-NHS hydrogel, mainly depending on the amination reaction of the NHS-activated AA with the amino groups in the skin tissue, and the strength of adhesion can also be enhanced via the formation of hydrogen bonds between the hydrogels and the tissue. However, the high concentration of AA-NHS will lead to a decrease in the concentration of AA precursors, which in turn will weaken the hydrogen bonding that links the tissue to the hydrogels. The hydrogels containing 10 or 15 mg mL^−1^ AA-NHS had the highest adhesive strength, which shows great potential in hemostatic applications by acting as a sealant/adhesive/hemostat to rapidly seal hemostasis.

### Hemocompatibility and Cytocompatibility of the Hydrogels

**Fig. 3:**
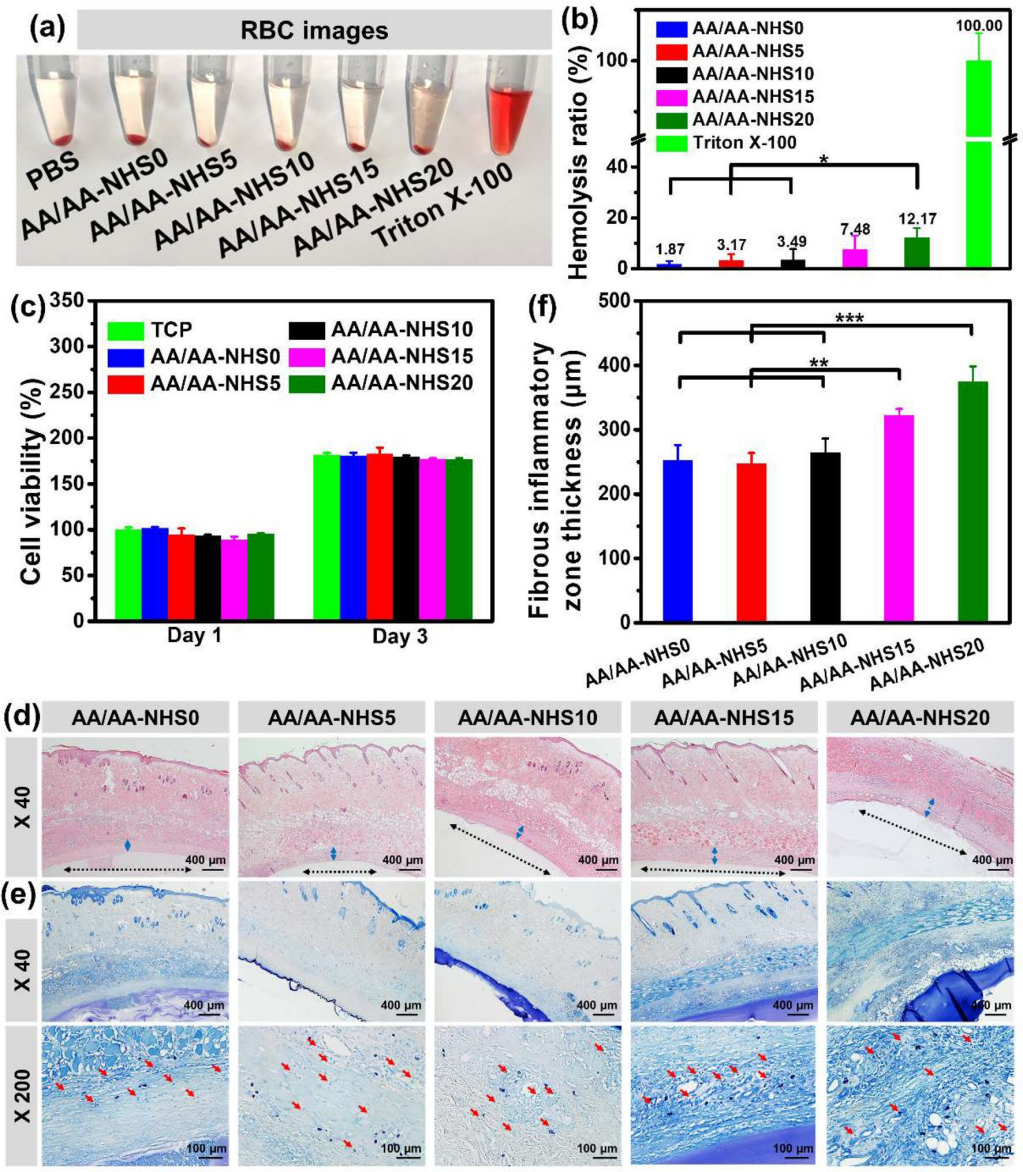
**a** Photographs of red blood cells (RBCs). **b** Hemolysis ratio (%) of the hydrogels (*n* = 3). **c** Quantitative analysis of L929 cell viability treated with the extracts of AA/AA-NHS hydrogels for 72 h at a concentration of 5 mg/mL. Representative images of **d** H&E-stained and **e** TB-stained sections of the subcutaneously implanted AA/AA-NHS0, AA/AA-NHS5, AA/AA-NHS10, AA/AA-NHS15, and AA/AA-NHS20 hydrogels with surrounding tissues. The blue arrows indicate fibrous inflammatory zone, and the black arrows indicate AA/AA-NHS hydrogel area, and the red arrows indicate mast cell. **f** Quantitative analysis of the thickness of the fibrous inflammatory zone. **P < 0.01, ***P < 0.005

A prerequisite of hydrogels used in adhesive hemostatic applications is good hemocompatibility. Thus, the hemocompatibility of the AA/AA-NHS hydrogels was evaluated by employing an in vitro hemolysis assay. The macroscopic color of all five hydrogels was light yellow, which was similar to that of the PBS group, while the color of the Triton X-100 group was bright red (Fig. 3a). The hemolysis ratios were 1.87, 3.17, 3.49, 7.48, and 12.17 for the AA/AA-NHS0, AA/AA-NHS5, AA/AA-NHS10, AA/AA-NHS15, and AA/AA-NHS20 hydrogels, respectively (Fig. 3b). The results showed significant hemolytic activity once the concentration of AA-NHS in the hydrogel was higher than 10 mg mL^−1^, while the hemolysis ratios in the AA/AA-NHS0, AA/AA-NHS5, and AA/AA-NHS10 hydrogel groups were within 5%, which is good for biomedical applications.

The cytocompatibility of the hydrogels was evaluated by coculturing L929 fibroblast cells with hydrogel extract at a concentration of 5 mg mL^−1^. The Alamar blue assay showed an increasing tendency from day 1 to day 3, which indicates good growth of the cells throughout the whole experiment. There was no obvious difference in the cell viability quantitative data between the hydrogel groups and the positive control group after co-incubation for 24 h. After 3 days of incubation, reduced cell proliferation was displayed in all hydrogel groups compared with the TCP group, but there was no difference in cell viability between the TCP and hydrogel groups, which illustrated that the hydrogel extracts did not leach cytotoxic content (Fig. 3c). In addition, most L929 cells were green and showed a spindle-like morphology after incubation with the extracts for 3 days, indicating the good cytocompatibility of the hydrogels (Fig. S4).

Besides, after co-incubation for 24 h, the hydrogel precursor solutions that were polymerized for 3, 6, or 9 min also exhibited acceptable cell viability (> 90%) compared with the TCP group (Fig. S5). In conclusion, all these results indicated the good in vitro cytocompatibility of these hydrogels.

The in vivo biocompatibility of the hydrogels was further evaluated by conducting a subcutaneous implant test. Hemoxylin and eosin (H&E) and toluidine blue (TB) staining were performed to investigate the host inflammatory response of the hydrogels. After implantation for 7 days, mild acute inflammatory responses were exhibited in all hydrogel groups, and the fibrous inflammatory zone thickness was similar around the AA/AA-NHS0, AA/AA-NHS5, and AA/AA-NHS10 hydrogel groups (Fig. 3d, f). The inflammatory response was also evaluated by performing TB staining to identify the mast cells around the hydrogel. The TB staining results showed that the number of mast cells over the surrounding tissues implanted with the hydrogels were similar to each other (Fig. S6), indicating that the acute inflammatory responses from the AA/AA-NHS hydrogels were as mild as those of the NHS-free group (Fig. 3e), and the acute inflammatory responses in AA/AA-NHS hydrogel groups was at an acceptable level compared with that of polycaprolactone (an FDA approved material) [52]. In conclusion, H&E and TB staining demonstrated the good in vivo biocompatibility of the hydrogels [27, 53, 54]. Therefore, the AA/AA-NHS10 hydrogel was chosen for further study due to its good hemocompatibility, cytocompatibility, and adhesive strength.

### In Vivo Hemostatic Performance of the Hydrogels

**Fig. 4:**
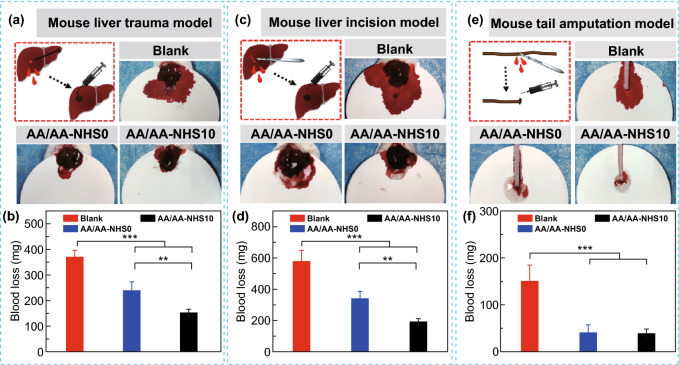
**a** Schematic representation of the mouse liver trauma model. **b** Quantitative data of blood loss (*n* = 6). **c** Schematic representation of the mouse liver incision model. **d** Quantitative data of blood loss (*n* = 6). **e** Schematic representation of the mouse tail amputation model. **f** Quantitative data of blood loss (*n* = 6). **P < 0.01, ***P < 0.005

As a novel adhesive hemostatic agent, AA/AA-NHS10 hydrogel achieves a good balance between adhesive strength and biocompatibility, which is verified from the results of the adhesive tests (Fig. 2i) and cytocompatibility (Fig. 3). Therefore, AA/AA-NHS10 hydrogel with good biocompatibility was selected to investigate the in vivo hemostatic performance and subsequent in vivo swine gastric bleeding/wound healing studies. The hemostatic behavior of the hydrogel was firstly detected by employing the mouse liver trauma model, the mouse liver incision model, and the mouse tail amputation model. In the mouse liver trauma model (Fig. 4a), compared with the blood loss in the control group (151.8 ± 14.9 mg), that in the hydrogel groups was much lower, while the hydrogel containing 10 mg mL^−1^ AA-NHS showed the lowest blood loss (370.7 ± 25.9 mg) (Fig. 4b). Furthermore, the hydrogel groups exhibited lower blood loss than the control group in both the mouse liver incision model (Fig. 4c, d) and the mouse tail amputation model (Fig. 4e, f). Besides, all the mouse in these three different bleeding models were still alive because the blood loss in each group was much lower than the critical blood volume loss [55]. The results in Fig. 4 suggest that the AA-NHS-containing hydrogel groups possess good hemostatic ability in these three hemorrhage models. The good in vivo hemostatic capability of the AA/AA-NHS hydrogel was mainly due to the adhesiveness of the hydrogels. The AA-NHS-containing hydrogel group had higher adhesive strength than the NHS-free group (Fig. 2i); therefore, these hydrogels could create a better seal on the wound, resulting in less blood flowing out. These results indicated that the injectable adhesive hydrogels are excellent hemostatic agent candidates for gastric wounds.

### In Vivo Hemostatic Performance of the Hydrogels in a Swine Gastric Bleeding Model

**Fig. 5:**
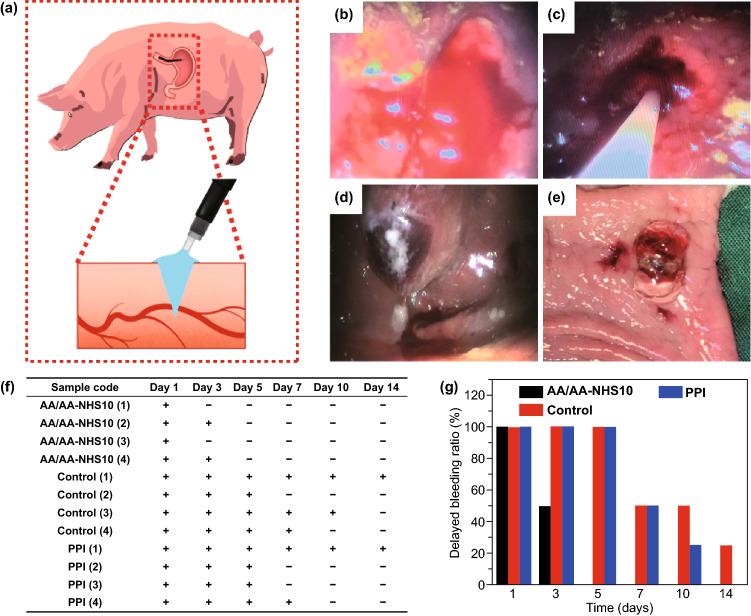
**a** Schematic representation of the investigation protocol diagram. **b** Swine gastric bleeding model. **c** The AA/AA-NHS10 hydrogel was sprayed to the bleeding point with a spray tube through a gastroscope. The hydrogel strongly adhered to the gastric wall and stopped bleeding by forming hydrogel films **d** in vivo and **e** in vitro. **f** Positive ratio of fecal routine test, “ + ” indicates blood in the stool in differently treated swine, while “−” indicates swine without blood in the stool. **g** The delayed bleeding ratio in the different treatment groups (*n* = 4)

Faced with the great challenge of effectively stopping rapid hemorrhage after endoscopic treatment, the most common clinical methods are intravenous injection of PPIs and hemostatic agents, endoscopic heater probe coagulation and hemoclips, and the oral intake of gastric mucosa protective agents. However, the effects of drug therapy on hemostasis and healing acceleration are limited, and delayed bleeding can occur in up to 20% of gastric cases. Endoscopic treatment of hemorrhages is an extremely challenging technique that needs substantial practice. Endoscopic heater probe coagulation or hemoclip treatment inevitably increases the risk of gastrointestinal perforation [56]. Therefore, we developed injectable self-healing adhesive hydrogels to rapidly stop the bleeding of gastric wounds. As bleeding is an inevitable consequence of endoscopic resection that occurs from the incised blood vessel either immediately or up to two weeks later, the hemostatic effects of the hydrogels were detected in a swine gastric bleeding model (Fig. 5a). After the posterior gastric wall and arterial rete were removed, continuous bleeding was observed (Fig. 5b). The AA/AA-NHS10 hydrogel was then immediately sprayed into the bleeding area using a spray tube through a gastroscope. Through gastroscopy, we found that the hydrogel strongly adhered to the gastric wall by forming a hydrogel film. The AA/AA-NHS10 hydrogel showed an immediate hemostatic function by stopping relevant hemorrhage within seconds through gelation on the bleeding focus, and no more blood came out of the wound (Fig. 5c–e). Fecal occult blood tests (FOBTs) were carried out using the colloidal gold method to investigate the hemostatic effects of the AA/AA-NHS10 hydrogel against delayed bleeding (delayed bleeding was defined as bleeding after an endoscopic operation). The results of the fecal routine test (Fig. 5f) showed that the positive ratio was significantly decreased in the AA/AA-NHS10 endosprayed swine group after 3 days of treatment compared with that in the esomeprazole-treated and control swine. The delayed bleeding ratio of swine in the different treatment groups was continuously observed, and the results in Fig. 5g show that this ratio in the hydrogel group significantly decreased after 3 days of treatment, and no obvious bleeding was found after treatment for 5 days. All of these results illustrate that AA/AA-NHS10 hydrogel endospray-treated swine were secured for hemostasis after the first hydrogel endospray and there was no need for a second treatment.

In the swine gastric bleeding model and gastric specimen, AA/AA-NHS10 hydrogel films stuck tightly to the gastric wall because of their adhesive capability, ensuring their effectiveness to stop acute arterial hemorrhage within seconds. The fecal routine test indicated that the hydrogel could prevent delayed bleeding more effectively than an intravenous injection of a PPI. As tension caused by gastric peristalsis, dilatation, and contraction would damage the hydrogel dressing, the rapid self-healing ability could repair these damages automatically and extend the service life, tensile strength, and strong adhesive force of the hydrogel. These abilities of the hydrogel contributed to isolation of the bleeding vessels from the natural acids of the stomach, thus preventing delayed bleeding. Furthermore, the aminocaproic acid in the hydrogel can effectively prevent delayed bleeding by inhibiting the activation of plasminogen for an extended service life. Therefore, these mechanisms of the AA/AA-NHS10 hydrogel facilitate the stoppage of acute arterial bleeding and prevent delayed bleeding in a swine gastric bleeding model.

### In Vivo Healing Promotion Effects of the Hydrogels in a Swine Gastric Wound Model

**Fig. 6:**
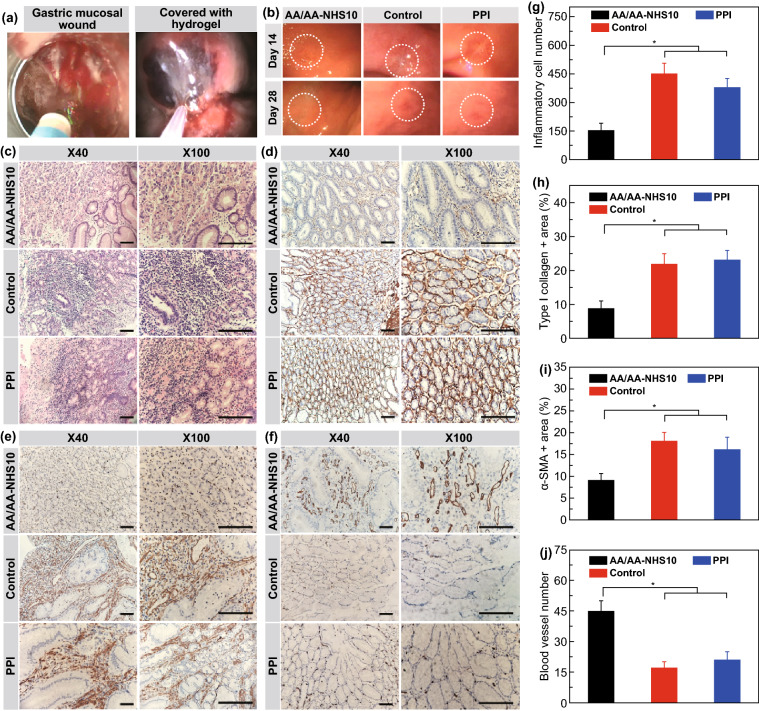
The wound healing performance of the AA/AA-NHS10 hydrogel in the swine gastric ESD model. **a** Investigation protocol diagram. **b** Healing status of the gastric wound on AA/AA-NHS10 hydrogel endospray-treated, esomeprazole-treated and control swine on days 14 and 28. The white circle indicates the wound area. **c** H&E staining and immunobiological staining of **d** type I collagen, **e** α-SMA, and **f** CD34 in wound tissue sections from AA/AA-NHS10 hydrogel endospray-treated, esomeprazole-treated and control swine after 28 days. Semi-quantification of the inflammatory cells in **g** H&E, **h** type I collagen, **i** α-SMA, and **j** micro-blood vessels in wound tissue sections from AA/AA-NHS10 hydrogel endospray-treated, esomeprazole-treated and control swine after 28 days (*n* = 4). Scale bar: 400 µm. *P < 0.05, **P < 0.01

In addition to blood coagulation, the gastric wound healing process after endoscopy resection included inflammation activation, tissue adhesiveness, angiogenesis, re-epithelialization, and ECM remodeling. Therefore, we next evaluated the impacts of AA/AA-NHS hydrogels on these processes using the swine ESD model. During the construction of the animal model, the gastric mucosal layer was resected within a diameter of 2 cm. The hydrogel was then sprayed into the gastric wound area using a spray tube through a gastroscope (Fig. 6a). All swine, including the PPI-treated group and the control group, survived the follow-up time of 28 days without showing any signs of physical impairment or systemic inflammation. The gastric wound surface was clearly closed after 14 days in the AA/AA-NHS hydrogel-treated swine compared with the PPI-treated and control swine. The resected mucosal layer had entirely healed after 28 days in the hydrogel-treated group (Fig. 6b). To detect the biological effects of the hydrogel, we further investigated the histological differences in gastric tissues among the 3 groups. Inflammatory cells were selected as an indicator to evaluate whether AA/AA-NHS hydrogels can change the inflammatory response and possibly accelerate the transition from inflammation to the proliferation phase [57]. The H&E staining results showed that the number of invading inflammatory cells in gastric wounds significantly decreased in AA/AA-NHS10-treated swine compared with PPI-treated and control swine. In addition, α-SMA and type I collagen were selected to further explore whether AA/AA-NHS hydrogels have the functions of accelerating wound healing and suppressing fibrosis. Immunohistochemistry (IHC) assays revealed that the expression levels of α-SMA and type I collagen in the gastric wounds were significantly suppressed by the AA/AA-NHS hydrogel, indicating that the hydrogel could suppress fibrosis during ECM remodeling. Microvessel density can be evaluated by immunohistochemistry staining of CD34 [58], and CD34 IHC staining showed that the number of blood vessels in the gastric wound significantly increased in the hydrogel-treated group compared with the control groups (Fig. 6c–j). These results suggested that the AA/AA-NHS hydrogel could promote gastric wound healing after endoscopic therapy by controlling inflammation and promoting ECM remodeling and angiogenesis. These hydrogels were even more effective than PPIs, which is the first-line treatment for post-endoscopic resection therapy and showed promising prospects for clinical applications.

The physical functions of hydrogels can protect gastric wound sites from erosion due to the natural acids of the stomach, isolate wound sites from bacterial infection, maintain a moist microenvironment, and allow for the presence of oxygen. These effects can facilitate AA/AA-NHS10 hydrogels to effectively promote gastric wound healing by controlling inflammation. Moreover, the aminocaproic acid in AA/AA-NHS10, as an antifibrinolytic agent, can inhibit plasmin activation by physical binding and prevent plasmin from binding to fibrin; additionally AA/AA-NHS10 cannot bind to the proteolytic site of plasmin, which allows the other role of plasmin as a growth factor activator to remain to promote wound healing [59]. Accordingly, the AA/AA-NHS10 hydrogel was also found to promote angiogenesis during wound healing, thus providing a favorable microenvironment to facilitate the ECM remodeling process. In addition, α-SMA and type I collagen expression were effectively inhibited by the hydrogel, which suggested that the AA/AA-NHS10 hydrogel can suppress fibrosis and prevent cicatricial stricture during wound healing. This mechanism may rely on the antiproteolytic effects of aminocaproic acid, which can stabilize the fibronectin matrix and facilitate the migration and adhesion of epithelial cells [60]. Therefore, these synergistic effects of AA/AA-NHS10 hydrogels significantly accelerated the process of gastric wound healing after endoscopic resection.

## Conclusions

We presented a series of injectable self-healing and adhesive AA/AA-NHS hydrogels for the treatment of gastric bleeding and wound healing after endoscopic treatment in a swine gastric wound model. The hydrogel was synthesized by a facile approach through the free-radical polymerization of AA and AA-NHS monomers under physiological conditions. The adhesiveness of the hydrogels was confirmed to be related to the AA-NHS concentration. The AA/AA-NHS hydrogels showed a suitable gelation time, efficient and repeatable self-healing properties, and good hemostatic properties. The good biocompatibility of the hydrogels was confirmed by the in vitro examination of coculture with L929 cells and hemolysis, and the in vivo of subcutaneous implant tests. Furthermore, AA/AA-NHS10 showed excellent therapeutic efficacy in in vivo gastric hemostasis and wound healing in a swine gastric hemorrhage/wound model by means of fecal routine tests, type I collagen deposition, α-SMA expression, and blood vessel formation. In summary, these injectable self-healing and adhesive AA/AA-NHS hydrogels are competitive candidates for gastric hemostasis and wound healing applications.

## Supplementary Information

Below is the link to the electronic supplementary material.

Supplementary Information1 (PDF 936 kb)

Supplementary Information1 (AVI 1453 kb)
