# Supplementary material for: Injectable Self-Healing Adhesive pH-Responsive Hydrogels Accelerate Gastric Hemostasis and Wound Healing
Source: Nanomicro Lett. 2021 Feb 27;13:80. doi: 10.1007/s40820-020-00585-0 (PMC8187506; doi:10.1007/s40820-020-00585-0)
Supplement: Supplementary file 1 — Supplementary Information1 (PDF 936 kb) [file 40820_2020_585_MOESM1_ESM.pdf]

Supporting information for

Injectable Self-Healing Adhesive pH-Responsive Hydrogels Accelerate Gastric Hemostasis and Wound Healing

Jiahui He[1, 2, #], Zixi Zhang [1, 2, 3, #], Yutong Yang[1], Fenggang Ren[4], Jipeng Li[5], Shaojun Zhu[6], Feng Ma[4], Rongqian Wu[4], Yi Lv[4], Gang He[2], Baolin Guo[1, 2, *], Dake Chu[1, *]

[1]Department of Gastroenterology, the First Affiliated Hospital of Xi'an Jiaotong University, Xi'an, P. R. China

[2]Frontier Institute of Science and Technology, and State Key Laboratory for Mechanical Behavior of Materials, Xi'an Jiaotong University, Xi'an, 710049, China Key Laboratory of Shaanxi Province for Craniofacial Precision Medicine Research, College of Stomatology, Xi'an Jiaotong University, Xi'an 710049, P. R. China

[3]Department of Dermatology, The First Affiliated Hospital of Xi'an Jiaotong University, Xi'an 710049, P. R. China

[4]National Local Joint Engineering Research Center for Precision Surgery and Regenerative Medicine and Surgical Engineering Research Center of Shaanxi Province, First Affiliated Hospital of Xi'an Jiaotong University, Xi'an, P. R. China

[5]Department of Experimental Surgery, Xijing Hospital, Fourth Military Medical University, Xi'an, P. R. China

[6]Department of Pathology, Tangdu Hospital, Fourth Military Medical University, Xi'an, P. R. China

[#]Jiahui He and Zixi Zhang contributed equally to this work

*Corresponding authors. E-mail: baoling@mail.xjtu.edu.cn (Baolin Guo), chudake@xjtu.edu.cn (Dake Chu)

S1 Materials and Methods

S1.1 Materials

6-aminocaproic acid (A) was purchase from Aladdin. Ammonium persulfate (APS) and sodium hydroxide (NaOH) were obtained from Sigma. All other materials or chemical reagents were used without further purification unless otherwise stated.

S1.2 Synthesis of AA and AA-NHS

AA was synthesized according to the reference [S1]. 19.8 g of 6-aminocaproic acid was dissolved in 120 mL of deionized water in an ice bath under vigorous stirring, and then, 6.6 g of NaOH was added to the solution. To this, 13.35 mL of acryloyl chloride in 22.5 mL tetrahydrofuran was added dropwise. The pH was maintained at

7.5-7.8 until the reaction was complete. The reaction mixture was then extracted with ethyl acetate. The clear aqueous layer was acidified to pH 3.0 and then extracted again with ethyl acetate. The organic layers were collected, combined, and dried over sodium sulfate. The solution was then filtered, concentrated, and precipitated in petroleum ether. Further purification was achieved by repeated precipitation and the product was lyophilized.

S1.3 Characterization

The FT-IR spectra of A and AA-NHS were recorded on a Nicolet 6700 FT-IR spectrometer (Thermo Scientific) in the 2000–800 cm^{-1} range.

The synthesized AA and AA-NHS were dissolved in deuterated DMSO, respectively. Then, the ^{1}H nuclear magnetic resonance (^{1}H NMR) (AVANCE III 400 MHz, Bruker) of AA and AA-NHS monomer were recorded at 25 °C.

After preparation of the desired concentration of AA and AA-NHS monomer solution and then mixed in a centrifuge tube (5 mL), the sol to gel transition at room temperature was visually observed by inverting the vials. The gelation time was determined when the mixture solution stopped flowing upon tube inversion in 60 s.

The swelling behaviors of the hydrogels at different pH were determined by the gravimetric method [S2]. The temperature was set at 37 ºC throughout the experiment to simulate body temperature. In detail, the completely gelled wet hydrogel with the same volume (500 μL) was firstly prepared. Then, the equilibrium swelling ratio (ESR) of the hydrogel at different pH was determined by immersing the as-prepared

hydrogel bulks in 20 mL PBS (0.01 M, pH 7.4) or artificial gastric juice (pH 2.0) in sealed vials. The hydrogel samples were taken out from PBS solution or artificial gastric juice and filter paper was used to remove the superficial water. Following that, the hydrogels were weighed until swelling equilibrium without external water at various times. The weights were recorded and the ESR was calculated using the following equation:

$$ESR = (W_t - W_0)/W_0 \times 100\% \qquad (S1)$$

where W_0 and W_t represented the initial weight of the wet hydrogels and the weight at the swollen state, respectively. The test was repeated three times.

The in vitro degradation tests were performed to investigate the degradation behavior of AA/AA-NHS hydrogels by immersing AA/A-NHS hydrogel bulks in PBS solutions. Besides, the hydrogel bulks with the same volume were immersed in artificial gastric juice (AGJ)/artificial intestinal juice (AIJ) at 37 °C to simulate the degradation behavior of AA/AA-NHS hydrogels in vivo. The detailed procedures of the degradation tests are as follows:

The hydrogel bulks with the same volume were put into 30 mL PBS/AGJ/AIJ solutions at 37 °C with shaking at 100 rpm. At each pre-set time interval, the samples were taken out. The hydrogel samples were lyophilized, and the weights were

recorded. The weight remaining ratio of AA/AA-NHS hydrogels was defined by the following equation:

$$\text{Weight remaining ratio of AA/AA-NHS hydrogel (\%)} = W_t / W_0 \times 100\% \quad \text{(S2)}$$

where W_t and W_0 are the lyophilized weight of the remaining hydrogels after degradation at different time points and the dry weight of the initial hydrogels, respectively. The test was repeated three times.

S1.4 Rheological Behavior of AA/AA-NHS Hydrogels

The rheological measurements of AA/AA-NHS hydrogels were determined by employing a TA rheometer (DHR-2). The storage moduli (G′) of AA/AA-NHS hydrogels was evaluated by a time sweep test with 1% constant strain and a constant frequency of 10 rad/s at 37 °C. 300 μL of the hydrogel polymer solutions were placed between 20 mm parallel plates with a gap of 1000 μm. Besides, to prevent water evaporation, the periphery of parallel plates was sealed by silicone oil.

S1.5 Self-healing Property of AA/AA-NHS Hydrogels

(1) **Quantitative self-healing test**

Self-healing test of AA/AA-NHS hydrogels was evaluated according to the publication [S3, S4]. AA/AA-NHS10 hydrogel samples with diameter of 20 mm and thickness of 1 mm were first prepared, and then the value of the critical strain region was recorded by performing the strain amplitude sweep method (γ from 1% to 2500%). Then the other hydrogel disks were used to evaluate the self-healing performance by carrying out an alternate step strain sweep test at a fixed angular frequency (10 rad/s). The amplitude oscillatory strains were switched from small strains (γ = 1.0%) to subsequent large strains (γ = 2000%), with 100 s for every strain interval and 5 cycles were carried out.

(2) **Macroscopic self-healing experiments of AA/AA-NHS hydrogels**

A circular mold with a diameter of 45 mm was prepared for macroscopic self-healing experiments. A glass baffle with a height of 15 mm was placed in the mold. Subsequently, 4 mL of crystal violet stained AA/AA-NHS10 hydrogel was then injected through a syringe to the left of the mold after polymerization while the hydrogel in right without crystal violet staining. After gelling for ten minutes, the self-healing behavior of resultant hydrogels was measured after different time intervals.

S1.6 Morphology of AA/AA-NHS Hydrogels

The lyophilized AA/AA-NHS hydrogels were sputtered with gold. Then the morphologies of the samples were investigated using a field-emission SEM (Quanta FEG 250, FEI). The average diameter of the hydrogels was determined by using the software ImageJ.

S1.7 Adhesive Strength Test of AA/AA-NHS Hydrogels

The adhesive ability of the hydrogels was evaluated according to the previously described method with some modification [S5]. The experiment was conducted by a lap-shear test and fresh porcine stomach was selected as an adherend. Briefly, the fresh porcine stomach was prepared into rectangle pieces with 1×3 cm^2 size and then immersed into PBS before use. After that, 100 μL AA/AA-NHS hydrogel polymer solutions were injected on the surface of porcine stomach then another adherend was placed on the hydrogel solution. The adhesive area was 10×10 mm^2. Subsequently, the porcine stomach was placed at 25 °C for 3 h. The adhesion properties were tested using the lap shear test on an Instron Materials Test system (MTS Criterion 43, MTS Criterion) equipped with a 50 N load cell at a rate of 5 mm min^{-1}. The test was repeated five times.

S1.8 Hemolysis Activity Assay of AA/AA-NHS Hydrogels

The hemolysis activity assay of AA/AA-NHS hydrogels was conducted to evaluate the blood compatibility of AA/AA-NHS hydrogels [S6, S7]. The erythrocytes were obtained by centrifuging (1000 rpm) of the mouse blood for 10 min. PBS was used to wash the obtained erythrocytes for three times, and then the purified erythrocytes were further diluted to a final concentration of 5% (v/v). After that, 500 μL of the erythrocytes were mixed with 500 μL of AA/AA-NHS hydrogel in 24-well culture plate. After placed at 37 °C for 1 h with a shaking speed of 100 rpm. Afterward, 500 μL of fresh PBS was added to each hydrogel contained microplate well. All the microplate well contents were centrifuged at 1000 rpm for 10 min to remove hemolysis-free erythrocytes. A volume of 100 μL of the supernatants was carefully transferred into a new 96-well clear plate. The absorbance of the solutions at 540 nm was read using a microplate reader (Molecular Devices). 0.1% Triton X-100 served as the positive control and DPBS served as the negative control.

Hemolysis ratio was calculated as following equation:

$$\text{Hemolysis ratio (\%)} = (OD_t - OD_n)/(OD_p - OD_n) \times 100\ \% \qquad \text{(S3)}$$

where OD_t, OD_n and OD_p were the absorbance values of samples, negative control (PBS) and positive control (Triton x-100), respectively. All the tests were carried out with 3 times of repetition.

S1.9 Cytocompatibility Evaluation of AA/AA-NHS Hydrogels

The cytocompatibility of the hydrogel was evaluated by co-culturing mouse fibroblast (L929) cells with the hydrogel's extract at a certain concentration [S8]. A series of sterilized AA/AA-NHS hydrogel extracts with a fixed concentration of 5 mg mL^{-1} were prepared by soaking each lyophilized AA/AA-NHS hydrogel sample with cell culture medium at 37 °C for 48 h. L929 cells were incubated at 37 °C in a humidified incubator containing 5% CO_2. The complete growth medium was consisted of DMEM, 10% fetal bovine serum, 1.0×10^5 U L^{-1} penicillin, and 100 mg L^{-1} streptomycin. 3000 L929 cells suspended in 100 μL of the complete growth medium were seeded in each well of a 96-well plate. After being cultured for 24 h, the medium

was changed into 100 μL of the sample's extracts. After being co-incubated for 24 h, the medium was removed and 10 μL alamarBlue® reagent in 100 μL complete growth medium were added into each well in the plate. The plate was incubated for 4 h in a humidified incubator containing 5% CO_2 at 37 °C. Next, 100 μL of the medium in each well was transferred into a 96-well black plate (Costar) and the microplate reader was used to read the fluorescence of each well to evaluate the cell viability. The live-dead cells were measured by a Live/Dead staining kit (Invitrogen) samples with cells were washed by PBS and stained with a Live/dead Viability/Cytotoxicity kit consulting the user guide. The cell images were observed under an inverted fluorescence microscope (IX53, Olympus).

For evaluating the cytocompatibility of AA/AA-NHS10 precursor solution, the precursor hydrogel solution after polymerizing for 3, 6, or 9 min was directly injected to L929 cells. After being co-incubated for 24 h, the medium was removed and 10 μL alamarBlue® reagent in 100 μL complete growth medium were added into each well in the plate. The plate was incubated for 4 h in a humidified incubator containing 5% CO_2 at 37 °C. Next, 100 μL of the medium in each well was transferred into a 96-well black plate (Costar) and the microplate reader was used to read the fluorescence of each well to evaluate the cell viability.

S1.10 In Vivo Biocompatibility of AA/AA-NHS Hydrogels

The in vivo biocompatibility of all the hydrogels were examined by injecting the hydrogel under the skin of SD rats. AA/AA-NHS hydrogels were retrieved at 7 days after being injected into the SD rats subcutaneously on the dorsum after sterilization. The hydrogels near tissue were removed, fixed with 4% paraformaldehyde for 24 h,

embedded in paraffin, and sectioned for further histological examination. All species were stained by the Hematoxylin–Eosin (H&E) and Toluidine blue (TB) staining methods to evaluate the inflammatory response of the hydrogels. The stained sections of each sample were examined by a microscope (Olympus, Japan) for evaluation of the tissue inflammatory reaction. All animal experiments were carried out in accordance with current guidelines for the care of laboratory animals and were approved by proper committee of Xi'an Jiaotong University.

S1.11 Hemostasis Performance of AA/AA-NHS Hydrogels

The hemostatic performance of AA/AA-NHS hydrogels was evaluated by employing the mouse liver trauma model, the mouse liver incision model, and the mouse-tail amputation model (Kunming mice, 30-35 g, female).

(1) **The mouse liver trauma model**

Briefly, a mouse was anesthetized and then fixed on a surgical corkboard. The liver of the mouse was exposed by abdominal incision, and serous fluid around the liver was carefully removed. A pre-weighed filter paper on a paraffin film was placed beneath the liver. Bleeding from the liver was induced using a 20 G needle with the corkboard tilted at about 30° and 300 μL of AA/AA-NHS hydrogel precursor solution that was

pre-polymerized at room temperature for 3 min was immediately applied to the bleeding site using the syringe. Fifteen minutes later, the weight of the filter paper with absorbed blood was measured and compared with a control group (no treatment after pricking the liver). All the measurements were carried out with 5 times of repetition.

(2) **The mouse liver incision model**

Briefly, a mouse was anesthetized and then fixed on a surgical corkboard. The liver of the mouse was exposed by abdominal incision, and serous fluid around the liver was carefully removed. A pre-weighed filter paper on a paraffin film was placed beneath the liver. Then, bleeding from the liver was induced by creating a wound (5 mm long, 2 mm deep) by using a scalpel and 300 μL of AA/AA-NHS hydrogel precursor solution that was pre-polymerized at room temperature for 3 min was immediately applied to the bleeding site using the syringe. Fifteen minutes later, the weight of the filter paper with absorbed blood was measured and compared with a control group (no treatment after pricking the liver). All the measurements were carried out with 5 times of repetition.

(3) **The mouse-tail amputation model**

A mouse was anesthetized and then fixed on a surgical corkboard. Fifty percent length of the tail was cut by surgical scissors. After cutting, the tail of the mouse was placed in air for 15 s to ensure normal blood loss. Then, 300 μL of AA/AA-NHS hydrogel precursor solution that was pre-polymerized at room temperature for 3 min was immediately applied to the bleeding site using the syringe. Fifteen minutes later, the weight of the filter paper with absorbed blood and hydrogel sample was measured and compared with a control group (no treatment after pricking the liver). All the measurements were carried out with 5 times of repetition.

S1.12 Hemostasis Performance and Wound Healing Behavior of AA/AA-NHS Hydrogels

The experiment of AA/AA-NHS hydrogels in a gastric hemorrhage model was performed at the animal experiment center of Xi'an Jiaotong University. In order to investigate the hemostasis effect and healing accelerating effect of AA/AA-NHS hydrogels, two experiments were conducted separately, 12 swine with body weight of 35 ± 2.1 kg (32.9–37.0 kg) were utilized in each experiment.

(1) For evaluating the hemostasis performance of AA/AA-NHS hydrogels, before the operating day, the swine were fasted but allowed full access to drinking water for 48 h for gastrointestinal preparation. In order to test the hemostasis effect of AA/AA-NHS hydrogels on the wound after endoscopic treatment or ulcer, gastric hemorrhage model was established by endoscope. An endoscope with water-jet function (CMD-90s, CETRO, Xian, China) was used for all procedures. And a virtual input/output (VIO) electrosurgical generator unit was used as a power source for electrical cutting. Specifically, after general anesthesia was induced following intratracheal intubation

on experimental swine, an endoscope was inserted into the swine's stomach and a hook knife (KD-620L; Olympus Medical, Tokyo, Japan) was inserted through the endoscope for electrical cutting. Then, mucosa and submucosa of posterior wall of gastric body was incised with the settings of electrosurgical generator to be pulse cut slow 40 W, until arterial rete was cut off and arterial bleeding was induced. For AA/AA-NHS10 hydrogel group, an endoscope with the diameter of a 23-gauge needle was employed to spray the hydrogel precursor solution that was pre-polymerized for 3 minutes at room temperature to the bleeding site. In control group, swine with gastric bleeding was intravenously injected with 40 mg of Esomeprazole (AstraZeneca DEL, USA) each day for 7 days. In negative control group, swine with artificial ulcer was untreated. Fecal occult blood tests (FOBTs) were performed using the colloidal gold method (Aibo occult blood detection reagent; Aibo Biomedical, Hangzhou, China) each day for 14 days. Specifically, a small amount of stool samples from experimental swine were collected. Then, samples were smeared in a test tube and immediately taken to the clinical laboratory. The assay was performed according to the product instructions as described before [S9]. The result was considered positive when both the detection line and the quality control line appeared.

The delayed bleeding ratio (%) in this study represents the percentage of swine with delayed bleeding to the total number of swine in the corresponding treatment group, which was determined by the following equation:

$$\text{The delayed bleeding ratio (\%)} = N_t / N_0 \times 100\% \quad \text{(S4)}$$

where Nt indicates the number of swine with blood in the stool and N_0 represents the total number of swine in each group.

For the investigation on the wound healing behavior of AA/AA-NHS hydrogels on the wound after endoscopic treatment or peptic ulcer, gastric wound and artificial ulcer

was induced following endoscopic submucosal dissection (ESD) procedures. After gastrointestinal preparation and general anesthesia was done on the experimental swine, an endoscope with a disposable distal transparent cap attachment (D-201-11804) on the tip was inserted into the swine's stomach and normal saline solution was injected into the submucosa of posterior wall of gastric body with a 23-g endoscopic injection needle (01841, Top Corporation, Tokyo, Japan) to elevate the mucosal layer. After that, a Dual knife (Olympus, KD-650L), with a 2-mm tip length (non-jet injector system) was used to incise mucosa. Then, an IT Knife (KD-611L; Olympus Medical, Tokyo, Japan) was used to dissect the roof mucosa to form a mucosal defect wound and artificial ulcer. For AA/AA-NHS10 hydrogel group, the ulcers were sprayed with the hydrogel precursor solution that was pre-polymerized at room temperature for 3 min. In PPI-treated group, swine with artificial ulcer was intravenously injected with 40 mg of Esomeprazole (AstraZeneca DEL, USA) each day for 14 days. In negative control group, swine with artificial ulcer was untreated. Euthanasia was administrated by intravenous injection of sodium pentobarbital in the ear vein after 28 days of healing and the stomach was extirpated. All experimental protocols have been approved by the ethics committee of Xi'an Jiaotong University.

S1.13 Histology and Immunohistochemistry

In hematoxylin-eosin (HE) assay, deparaffinized sections were immersed in hematoxylin solution for 8 min, followed by washing in double distilled water for 1 min. After dissimilation using 1% of hydrochloric acid ethanol for 8 s and washing in double distilled water for 1 min, the sections were soaked in 1% dilute ammonia for 30 s to turn blue. After another wash in double distilled water for 1 min, the sections were stained by 0.5% eosin solution for 2 min. Then 95% ethanol I and II were used for dehydration with 2 min each time, followed by absolute ethanol I and II for 2 min, respectively. The sections were cleared using xylene I, II and III for 2 min, respectively. Neutral resins were added in the section which was then sealed using a cover slide for mounting.

Immunohistochemistry assay was performed using the avidin–biotin–peroxidase method. All histological sections were deparaffinized in xylene and dehydrated through a gradient concentration of alcohol before endogenous peroxidase activity was blocked with 0.5% H_2O_2 in methanol for 10 min. Then, nonspecific binding was blocked. Sections were incubated with mouse anti- type I collagen, α-SMA, and CD34 (1:100, Maixin, Fouzhou, China) in phosphate-buffered saline (PBS) at 4 ºC overnight in a moist box. Negative controls were performed by replacing the primary antibody with pre-immune serum. Biotinylated anti-mouse IgG (1:400, Sigma) was incubated with the sections for 1 h at room temperature and detected with streptavidin–peroxidase complex. The brown color indication of peroxidase activity was obtained by incubating with 0.1% 3, 3-diaminobenzidine (Sigma) in PBS with 0.05% H_2O_2 for 5 min at room temperature. Appropriate positive and negative controls were included in each run of immunohistochemistry.

For the semi-quantification of immunohistochemistry assay, the number of the

inflammatory cells were counted in an area of 500 x 500 μm by using the software ImageJ. Microscope fields under high magnification (×100) with moderate or strong type I collagen or α-SMA immunostaining intensity were classified as positive (+) area, whereas fields with absent or weak immunostaining were classified as negative (-) area. Blood vessels with yellow or brown CD34 stained vascular endothelial cells under a high magnification (×100) field were counted for blood vessel number.

S2 Results and Discussion

Table S1 Parameters and gelation time of AA/AA-NHS hydrogels

Samples	AA (mg)	AA-NHS (mg)	Bis (mg)	APS (mg)	TEMED (μL)	Gelation time (s)
AA/AA-NHS0	100	0	0.5	5	1	~300
AA/AA-NHS5	95	5	0.5	5	1	~330
AA/AA-NHS10	90	10	0.5	5	1	~350
AA/AA-NHS15	85	15	0.5	5	1	~400
AA/AA-NHS20	80	20	0.5	5	1	~500

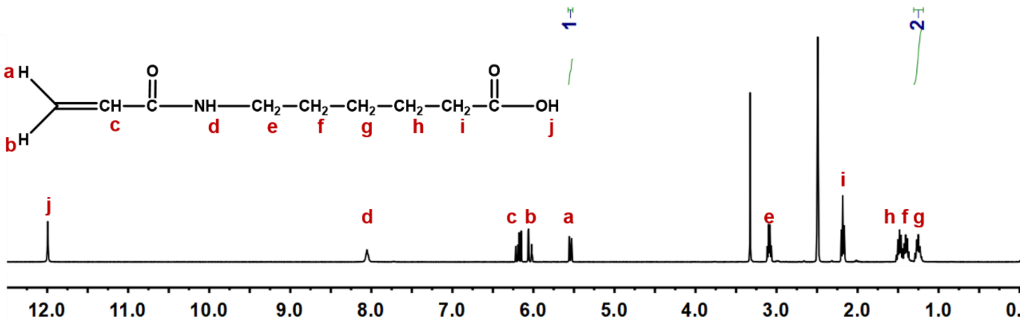

Fig. S1 ^{1}H NMR spectra of AA

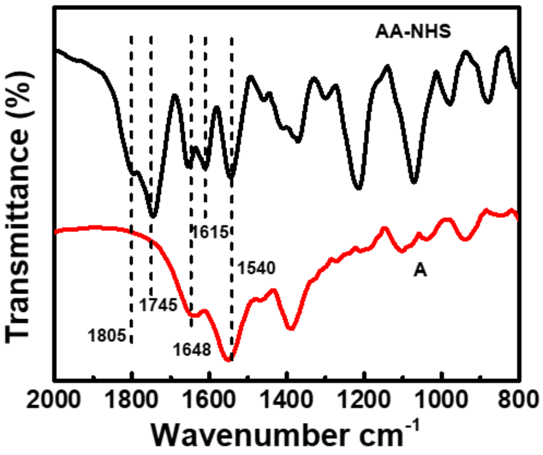

Fig. S2 FT-IR spectra of the synthetic monomer

In FT-TR curve of A, the characteristic peak in 1540 cm^{-1} was due to the N-H bending vibrations coupled to C-N stretching vibrations, while the peak in 1648 cm^{-1} was attributed to the stretching vibrations of C=O groups in A. The FT-TR curve of AA-

NHS showed all these characteristic peaks. Besides, compared with the FT-IR spectra of A, the FTIR spectra of AA-NHS showed a significant enhancement at the peak of 1735 cm$^{−1}$ which is attributed to conjugated carbonyl groups. And the peak at 1615 cm$^{−1}$ was associated with -C=C- of AA-NHS.

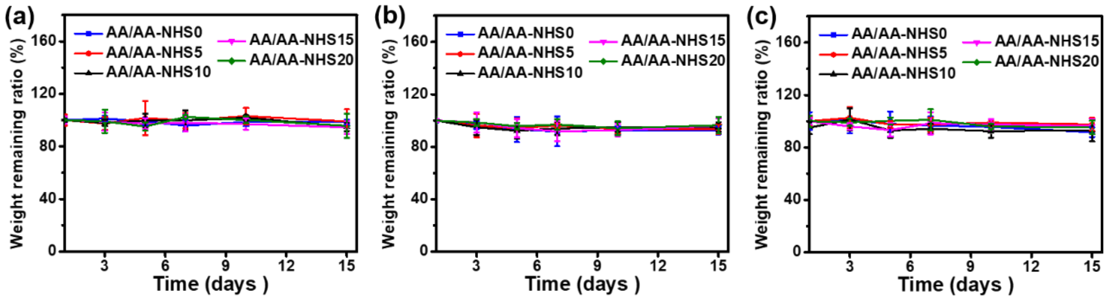

Fig. S3 In vitro degradation profiles of AA/AA-NHS hydrogels in PBS (**a**), artificial gastric juice (**b**), and artificial intestinal juice (**c**)

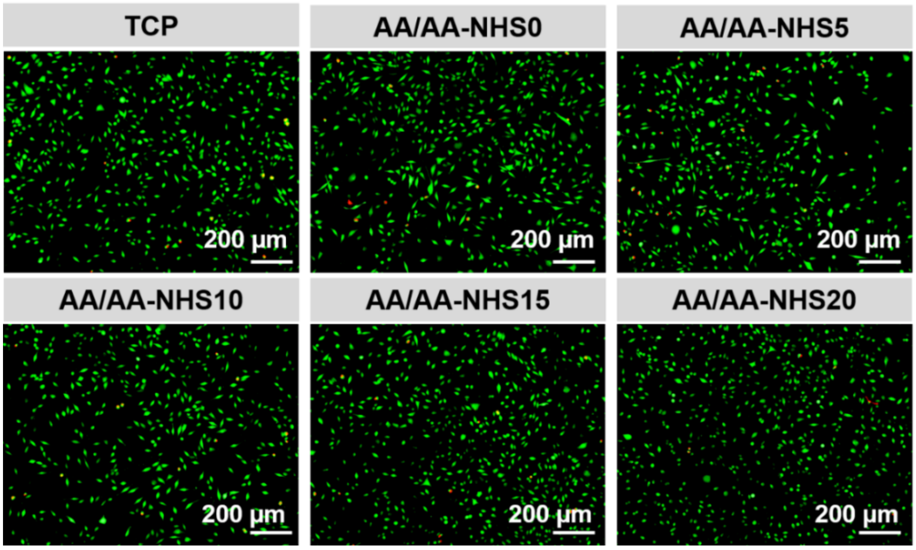

Fig. S4 LIVE/DEAD staining of L929 cells after incubated with the extracts for 3 days

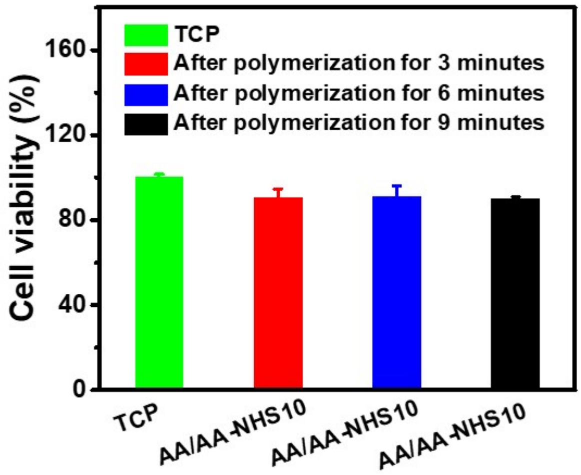

Fig. S5 Cell viability of AA/AA-NHS10 hydrogel by injecting AA/AA-NHS10 hydrogel precursor solution directly to L929 cells

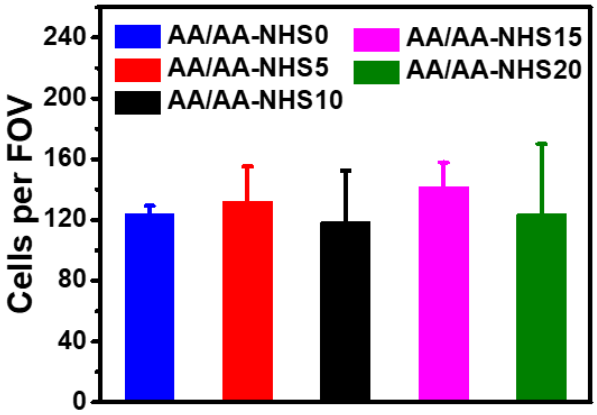

Fig. S6 Mast cell number per field of view (FOV) of AA/AA-NHS hydrogels at high magnification (×200). Samples were harvested after 7-day implantation

Supplementary References

[S1] A. Phadke, C. Zhang, B. Arman, C.-C. Hsu, R.A. Mashelkar et al., Rapid self-healing hydrogels. Proc. Natl. Acad. Sci. USA **109**, 4383 (2012). https://10.1073/pnas.1201122109

[S2] Z. Zhang, L. Chen, C. Zhao, Y. Bai, M. Deng et al., Thermo- and pH-responsive HPC-g-AA/AA hydrogels for controlled drug delivery applications. Polymer **52**, 676-682 (2011). https://doi.org/10.1016/j.polymer.2010.12.048

[S3] J. Qu, X. Zhao, Y. Liang, T. Zhang, P.X. Ma et al., Antibacterial adhesive injectable hydrogels with rapid self-healing, extensibility and compressibility as wound dressing for joints skin wound healing. Biomaterials **183**, 185-199 (2018). https://doi.org/10.1016/j.biomaterials.2018.08.044

[S4] B. Guo, J. Qu, X. Zhao, M. Zhang, Degradable conductive self-healing hydrogels based on dextran-graft-tetraaniline and N-carboxyethyl chitosan as injectable carriers for myoblast cell therapy and muscle regeneration. Acta Biomater. **84**, 180-193 (2019). https://doi.org/10.1016/j.actbio.2018.12.008

[S5] Y. Liang, X. Zhao, T. Hu, Y. Han, B. Guo, Mussel-inspired, antibacterial, conductive, antioxidant, injectable composite hydrogel wound dressing to promote the regeneration of infected skin. J. Colloid Interf. Sci. **556**, 514-528 (2019). https://doi.org/10.1016/j.jcis.2019.08.083

[S6] F. Cheng, C. Liu, X. Wei, T. Yan, H. Li et al., Preparation and characterization of 2,2,6,6-tetramethylpiperidine-1-oxyl (TEMPO)-oxidized cellulose nanocrystal/alginate biodegradable composite dressing for hemostasis applications. ACS Sustain. Chem. Eng. **5**, 3819-3828 (2017). https://10.1021/acssuschemeng.6b02849

[S7] Y. Huang, X. Zhao, Z. Zhang, Y. Liang, Z. Yin et al., Degradable gelatin-based IPN cryogel hemostat for rapidly stopping deepnoncompressible hemorrhage and simultaneously improving wound healing. Chem. Mater. **32**, 6595-6610 (2020). https://10.1021/acs.chemmater.0c02030

[S8] L. Zhang, L. Wang, B. Guo, P.X. Ma, Cytocompatible injectable carboxymethyl chitosan/N-isopropylacrylamide hydrogels for localized drug delivery. Carbohyd. Polym. **103**, 110-118 (2014). https://doi.org/10.1016/j.carbpol.2013.12.017

[S9] Q. Wen, K. Liu, W. Yue, S. Shao, S. Zhang et al., Clinical significance of positive fecal occult blood test in neonates. Sci. Rep. **9**, 17898 (2019). https://10.1038/s41598-019-54511-5
